# The Concept of Evenness/Unevenness: Less Evenness or More Unevenness?

**DOI:** 10.1007/s10441-021-09429-9

**Published:** 2021-12-10

**Authors:** Hans-Rolf Gregorius, Elizabeth M. Gillet

**Affiliations:** 1 Forstgenetik und Forstpflanzenzüchtung, Fakultät Forstwissenschaften und Waldökologie, Universität Göttingen, Büsgenweg 2, 37077 Göttingen, Germany; 2 Institut für Populations- und ökologische Genetik, Am Pfingstanger 58, 37075 Göttingen, Germany

**Keywords:** Concept of evenness, Functional evenness, Unevenness, Evenness index, Type representation, Diversity index, Abundance, Representation distribution, Variable difference, Neighborhood evenness, Variational evenness, Dispersion evenness

## Abstract

While evenness is understood to be maximal if all types (species, genotypes, alleles, etc.) are represented equally (via abundance, biomass, area, etc.), its opposite, maximal unevenness, either remains conceptually in the dark or is conceived as the type distribution that minimizes the applied evenness index. The latter approach, however, frequently leads to *conceptual inconsistency* due to the fact that the minimizing distribution is not specifiable or is monomorphic. The state of monomorphism, however, is indeterminate in terms of its evenness/unevenness characteristics. Indeed, the semantic indeterminacy also shows up in the observation that monomorphism represents a state of pronounced discontinuity for the established evenness indices. This serious conceptual inconsistency is latent in the widely held idea that evenness is an independent component of diversity. As a consequence, the established evenness indices largely appear as indicators of relative polymorphism rather than as indicators of evenness. In order to arrive at consistent measures of evenness/unevenness, it seems indispensable to determine which states are of maximal unevenness and then to assess the position of a given type distribution between states of maximal evenness and maximal unevenness. Since semantically, unevenness implies inequality among type representations, its maximum is reached if all type representations are equally different. For given number of types, this situation is realized if type representations, when ranked in descending order, show equal differences between adjacent types. We term such distributions “stepladders” as opposed to “plateaus” for uniform distributions. Two approaches to new evenness measures are proposed that reflect different perspectives on the positioning of type distributions between the closest stepladders and the closest plateaus. Their two extremes indicate states of complete evenness and complete unevenness, and the midpoint is postulated to represent the turning point between prevailing evenness and prevailing unevenness. The measures are graphically illustrated by evenness surfaces plotted above frequency simplices for three types, and by transects through evenness surfaces for more types. The approach can be generalized to include variable differences between types (as required in analyses of functional evenness) by simply replacing types with pairs of different types. Pairs, as the new types, can be represented by their abundances, for example, and these can be modified in various ways by the differences between the two types that form the pair. Pair representations thus consist of both the difference between the paired types and their frequency. Omission of pair frequencies leads to conceptual ambiguity. Given this specification of pair representations, their evenness/unevenness can be evaluated using the same indices developed for simple types. Pair evenness then turns out to quantify dispersion evenness.

## Introduction

There is general agreement on the concept of evenness as far as its one extreme of complete evenness is concerned. The concept is built on the representation of types in collections of objects, and it is oriented at the degree to which the types are represented equally. Types could be alleles or genotypes represented by their frequencies in populations, species represented by their abundances in communities, crop varieties represented by the area they cover or the biomass they yield in cultivation, etc. Hence, the focus is set on the representation of types but not on their numbers. This contrasts with common notions of diversity which comprise both numbers of types and their representations.

The latter explains the widespread habit to conceive diversity as combining number of types with the evenness of their representations. Quoting Hurlbert (1971, and further citations in this paper), “Species diversity is a function of the number of species present (species richness or species abundance) and the evenness with which the individuals are distributed among these species (species evenness or species equitability)”. The conceptual demands of the evenness notion on diversity measures was operationally specified as “transfer of abundance” (or principle of transfers) by Patil and Taillie (1982) and reformulated as the evenness criterion by Gregorius (2010): “diversity never decreases as the difference in frequency between two types decreases while the sum of their frequencies remains the same”). Strictly speaking, it is this criterion (further generalizations can be found in Grabchak et al. (2016)) that justifies the central conception of evenness as a component of diversity and allows transformation of each diversity measure into an “effective number” of types.

Other approaches to measuring evenness abandon the diversity concept altogether and turn directly to measures of distance of observed from ideal type distributions, where the ideal is defined by a uniform distribution (all type representations equal). These approaches are chiefly motivated by problems encountered with diversity-based evenness indices that are due to the assessment of distributional characteristics and statistical inestimability of indices [Pielou (1969, p. 234); Peet (1975, p. 497); Gregorius (1990); Bulla (1994)]. Bulla even reverses the relationship between diversity and evenness by recommending the product of his evenness measure with the number of types as a measure of diversity.

In all of the above-addressed work, the focus is set on complete evenness, and deviations from this ideal structural state are quantified in terms of normalized measures of diversity or distances of the observed type distribution from the ideal state. The smaller the values of the respective measures become, the larger the incongruence with the ideal state is scored. The structural characteristics of the type distributions which realize the minimal evenness, if they exist, could then be viewed to provide in some sense an idea of the absence of evenness. Yet, conceptual specifications of this idea are rarely, if ever, pondered. This is unfortunate, since it deprives us of any attempts to associate the absence of evenness with relevant ecological or evolutionary processes.

As a first step, common methods of quantifying evenness will therefore be checked for consistency of their lower bounds with notions of the absence of evenness. Remaining inconsistencies will be treated by turning from the absence of evenness to a concept of unevenness that is based on the specification of desirable structural characteristics of type representations. The measurement of evenness will then be designed to cover the continuum between complete evenness and complete unevenness. Herewith, *the leading thought is that less evenness is not the same as more unevenness unless maximal unevenness is as definitely defined as maximal evenness is.* As a consequence, the notion of maximum unevenness must be purely conceptually defined as is true for the notion of maximum evenness.

To appreciate the wide scope of applications, it is useful to consider that type representations are not just limited to the above-mentioned features but may also reflect relations among community members such as smallest or average difference of a type from other types in the community, as are applied in work on functional diversity. Several measures will be introduced in order to allow choices to be made according to intuitive access or data structure and to encourage adaptation of one’s own models.

The recently increasing interest in functional traits and their variation (commonly though improperly referred to as “functional diversity”) chiefly focuses on characteristics of the distribution of trait differences (however measured) in collections of organisms (especially communities or populations). In this context, the entities of consideration can be of different kind, such as individuals, types (species, genetic types, etc), and also pairs of those. The latter entities, pairs, are especially relevant in the assessment of functional variation in that pair differences and occurrence frequencies of pair types jointly determine functional relations among the members of communities. They will receive special consideration in a separate section which offers a conceptual solution for the measurement of functional evenness that avoids the shortcomings of currently favored indices.

## Established Methods of Measuring Evenness

As was mentioned before, the established indices of evenness can be distinguished into diversity-based and distance-based methods, both of which assume their respective maximal values (usually 1) only for uniform type representations. In the following, a brief demonstration will be provided of the distributional characteristics that can be associated with index values below the maximum and particularly as the values approach their lower bounds. The results will be discussed with respect to their compatibility with the basic conceptual requirements imposed on the indices as well as their statistical implications.

Throughout this paper, the relative representations $q_i$ of *s* types are assumed to be ranked in descending order such that $q_1\ge q_2\ge \ldots \ge q_s>0$ with $\sum _{i=1}^s q_i=1$. Uniform distributions of *s* types, i.e., $q_1=q_2=\ldots =q_s=1/s$, will be referred to as “plateaus” of length *s*. Whenever *s* is specified, the stipulation implies that $q_i=0$ for all $i>s$.

Let us start with the probably most frequently applied diversity index, Simpson’s index (Simpson 1949). This index is used in different versions, among which the probability of sampling with replacement two different types, i.e., $1-\sum _{i=1}^s q_i^2$, is usually preferred for both intuitive and conceptual reasons. The effective number of types involved in this index equals $1/\sum _{i=1}^s q_i^2$, and it appears as one of the family $N_a:=(\sum _{i=1}^s q_i^a)^{\frac{1}{1-a}}$ of diversity indices derived by Hill (1973) as effective numbers of Rényi’s family of entropy measures (Rényi 1961) (effective Simpson number = $N_2$; the limit for $a\rightarrow 1$ exists and equals $N_1=\exp (-\sum _{i=1}^s q_i\cdot \ln q_i)$). The characteristic of $N_a$ as an effective number of types becomes apparent from $N_a=s$ for *s* equally represented types, irrespective of the parameter *a*.

The by far most common transformation of the diversities $N_a$ into an evenness measure is $N_a/s$ for *s* types (for various variants of this family of measures, see e.g. Table 2 in Tuomisto (2012) or Table 1 in Kvålseth (2015)). The conceptual inconsistency of this measure shows up directly when considering a series of type distributions with constant number *s* of types, along which all representations are positive and tend to zero with the exception of the first. A non-trivial example is provided by models of resource apportioning that follow a finite geometric progression with the distribution

1 $$\begin{aligned} q_i=\frac{x^i\cdot (1-x)}{x\cdot (1-x^s)}=x^{i-1}\cdot {\frac{1-x}{1-x^s}}\ , \quad i=1,\ldots ,s \end{aligned}$$

where $0<x<1$ for the parameter *x*. Applying L’Hôpital’s rule, one obtains as the limit for $x\rightarrow 1$, $q_i=1/s$ for $i=1,\ldots ,s$ and thus a uniform distribution for *s* types. Moreover, as $x\rightarrow 0$, one obtains at the limit $q_1=1$ and $q_i=0$ for $i>1$. At the same time $N_a\rightarrow 1$, in accordance with the limiting distribution consisting of a single type (monomorphism). The evenness or unevenness state of monomorphism is, however, unresolved unless declared otherwise (such as “uniformity” by extending the concept from multiple types to a single type). Thus $N_a/s\rightarrow 1/s$ for all *s*, even though the limiting distribution always is the same (consisting of a single type). Thus the same distribution receives different index values.

This discrepancy can be avoided by transforming $N_a/s$ into $(N_a-1)/(s-1)$, so that one obtains an index that varies between 0 and 1 for ${s>1}$ [see e.g. Jost (2010), Eq. (3)]. This index would converge to 0 as $N_a$ tends to 1 and *s* (the number of types with positive representation) remains greater than one. Of course, at the limit the index is not defined, since ${s=1}$ holds there. Moreover, since the index is supposed to measure evenness, a value of 0 should indicate the “absence” of evenness, and the associated limiting state would be monomorphism. One would therefore be obliged to consider monomorphism as a state of the absence of evenness, even though otherwise the evenness state of monomorphism is considered to be unresolved.

The discrepancy indeed has practical relevance, for example in population genetics, where so-called “minor polymorphisms” (Lewontin 1974) resemble geometric progressions (Eq. 1) for *x* close to zero in that they consist of a single dominant allele and a number of rare alleles. More frequently, however, minor polymorphisms are found to resemble the form

2 $$\begin{aligned} q_1=1-(s-1)\cdot x/s, \quad q_i=x/s\quad \text {for}\ i=2,\ldots ,s\ \text {and}\ 0\le x\le 1 \end{aligned}$$

(L-shaped distribution), to which the above deliberations apply identically for small *x*. L-shaped distributions are a special case of mixtures of distributions as are typical for models of migration in population genetics or community ecology. Section 3.2 gives a geometric illustration of L-distributions along abundance transects. For more examples of type distributions and their underlying statistical models, see e.g. Sect. 2.2 in Heip et al. (1998).

The pitfall of normalizing indices by numbers of types was pointed out long ago. For example, Pielou (1969, p. 234) noted that evenness measures that are normalized in this way cannot be estimated. Obviously, even for large sample sizes, the existence of rare types in the sampled community may cause only small changes in the estimate of $N_a$ when sampling is repeated, but the number of types may change noticeably.

Hill (1973) was already aware of this drawback. He suggested an alternative by making use of the fact that $N_a\le N_b$ for $a>b>0$ with equality only for uniform distributions. His evenness index $N_a/N_b$ does not seem to be used, however. One of the reasons is probably the arbitrariness in the choice of levels *a* and *b* [see e.g. Ricotta (2003)]. Another reason might be that there is no definite greatest lower bound for $N_a/N_b$ which could be associated with structural characteristics of type distributions that indicate lowest evenness. In addition, the index also assumes its maximal value of 1 for monomorphism, which suggests that monomorphism is a state of complete evenness, as opposed to its previous characterization as the “absence” of evenness. Again one arrives at inconsistent conclusions.

The conceptual inconsistency of the index $N_a/s$ applies analogously to the occasionally used index of evenness suggested by Bulla (1994). The index is based on a measure of distance between a distribution of *s* types and the corresponding uniform distribution of these *s* types. The distance measure is $d=\frac{1}{2}\cdot \sum _{i=1}^s|q_i-1/s|$, which relates to the well-known Manhattan distance between the distribution *q* and the uniform distribution. It has a least upper bound of $d=1-1/s$ for given *s* that is reached if *q* represents a single type (monomorphism). Bulla’s evenness index can then be stated in the form $1-d/(1-1/s)$. It is immediately realized that the conceptual inconsistency demonstrated above for the diversity-based method $N_a/s$ (or $(N_a-1)/(s-1)$) of quantifying evenness applies identically to Bulla’s index (also see Ricotta et al. (2014)). The same holds when *d* is replaced by any other appropriate distance measure between distributions [Chao and Ricotta (2019)].

To avoid this problem, Gregorius (1990) suggested that the distance be minimized between the observed type distribution and all possible uniform type distributions. The minimization removes the explicit dependence of evenness measurement on the number of types, which is the major cause of the conceptual shortcoming of the common measures. In fact, assessment of the evenness of a type distribution should by basic perception not be a matter of number of types but rather only of their representations. While the distance minimization approach apparently realizes this principle, it can be shown that for given number of types the greatest lower bound (infimum) of the minimal distances increases with the number of types (Gregorius 1990). Yet, the characteristics of the type distributions that realize or become arbitrarily close to the respective lower bound again remain unspecified.

In other cases, the lower bound of a measure can be determined together with the distribution for which it is realized. This requires specification of additional conditions under which this can be achieved. Among these conditions is first of all that the representations of types remain properly positive as the lower bound is approached and finally realized. The condition is mandatory for retaining the number of types, since otherwise the number of types to be considered would be successively reduced as more and more types reach zero representation. Moreover, the condition implies that collections are of finite size, since for infinite size, the relative type abundances can approach values of zero arbitrarily closely without changing the number of types. Consequently, in a finite collection, the lowest representation of a type is reached if it occurs only once.

The situation where all but one of the types occur only once (of course the number of types in a collection is not allowed to exceed the collection size) is referred to as maximal unevenness by Bulla (1994) and Kvålseth (2015), since their indices are minimized under these conditions. The distribution of maximal unevenness therefore is L-shaped (Eq. 2), and it is conditional on two parameters, the number of types (*s*) and the lower threshold abundance for type presence (*x*). Essentially, this idea of a state of maximal unevenness underlies all of the common measures of evenness, though this is rarely stated explicitly [see the compilation of Scheiner (2019)]. In fact, Jost (2010) and more recently Chao and Ricotta (2019) addressed monomorphism as a conceptual criterion for maximal unevenness.

Conceptual reasoning why this situation specifies maximal unevenness, other than that it reaches the lower bound of specific evenness measures, is not provided. Both authors (Bulla and Kvålseth) do admit that their specification of maximal unevenness is problematic when considering increasing collection size. In fact, their reluctance can chiefly be ascribed to the above-mentioned problem of conceptual inconsistency. It also recognizes the problem of “inestimability of evenness”, in that when samples are taken from base collections of effectively infinite size, the true state of maximal unevenness would be the presence of only a single type, but the assessment of evenness in terms of complete unevenness is then questionable.

In the approach of Jost (2010), it is assumed that all components involved in the evenness notion are specified in terms of numbers equivalents (effective numbers), among which diversity (with emphasis on Hill numbers) takes a central position. The number of types (richness) is conceived to result from independent combination (in form of a product) of diversity and “some other independent quantity *X*” (also named “inequality factor”), which is concluded in the further analysis to be interpretable as a measure of unevenness that is equal to the multiplicative inverse of the common index $N_a/s$ of evenness, i.e. $X=s/N_a$. In this approach, the notions of evenness and unevenness are thus divided into different measures rather than being reflected in a single measure. Moreover, *X* has no upper bound, so that ideas of maximum unevenness are without substance. The above-mentioned problem of conceptual inconsistency remains.

In essence, it appears that the common indices proposed for the quantification of evenness [see e.g. Table 1 in Kvålseth (2015) and Table 1 in Chao and Ricotta (2019)], and especially those normalized to range within the unit interval, indicate complete evenness for values of 1 and indicate effective monomorphism for values close to 0. This observation is reminiscent of ideas of concentration of overall mass to a single type, for which a value of 0 indicates complete concentration and a value of 1 indicates equal distribution of mass over types, akin to the Gini index used in economics. Alternatively, in ecology the term “dominance” is used to refer to the same situation based on relative species abundances in which one or a few species dominate the species spectrum by their abundance [for a more detailed study, see Fung et al. (2015)]. The common evenness indices might therefore just as well be referred to as inverse indices of concentration or as inverse indices of dominance. Indeed, both concentration and dominance decrease as types become more evenly distributed.

An even more comprehensive description can be arrived at if one realizes both extremes of the common indices in terms of polymorphism. In this context, the minimal and maximal values indicate monomorphism and full polymorphism, respectively. Herewith “full polymorphism” is to be understood in the light of the limit set by the number of types occurring in the collection, so that the polymorphism is “full” in the sense that all types are equally abundant. From this perspective, the common approaches to measuring evenness are actually revealed to be characteristic of *measures of relative polymorphism*.

This ambivalence calls for a novel kind of approach to the measurement of evenness that involves a consistent concept of unevenness. Since the notion of evenness itself is unaffected, the questions to be treated are the same as before, with the difference that unevenness receives the significance which it initially was arguably intended to have.

## Concepts of Un-evenness

It has become clear by now that most of the shortcomings of the common measures of evenness go back to a disregard of (1) distribution characteristics (especially rare types) that lead to discontinuous transitions from polymorphism (more than one type with positive representation) to monomorphism, and (2) specification of the characteristics of type distributions that realize or come close to the greatest lower bounds (infima) of the respective index. The latter calls to attention the concept of unevenness that is thought to appear as small index values and the associated idea of low evenness. Simply conceiving of ever smaller values of the established measures of evenness as increasing unevenness is conceptually not justified.

When the opposite of evenness is to be characterized, the challenge is to define complete unevenness as the analogue and counterpart of complete evenness. Apparently, the concept of the analogue is not as obvious as the concept of complete evenness. Though various approaches are conceivable, it seems compelling to *conceive of unevenness as the negation of evenness and thus the entailment of inequality of type representations in the first place*. Following this, the question is as to the existence and structure of a state of maximal inequality in type representations. Herewith it must be taken into consideration that this state has to be specified for type distributions and thus for relative type representations that sum up to 1.

Maximization of inequality in representations is therefore difficult to envision without suitable ordering of the representations, such as the presently used ranking in descending order. Here it becomes immediately clear that simply enlarging differences in representation between individual objects may not increase the overall inequality, since it may rather increase equality between the representations of other types. In its extreme form, this occurs as the concentration of mass to one or a few types increases, which, in turn, entails the above-argued conceptual inconsistency. Consequently, overall inequality in representation can only be enlarged by distributing the differences between types as equably as possible. In other words, *unevenness should increase as all types become equally differently represented.*

Because of the uni-dimensionality of representations and the linear ordering implied by their ranking, maximal unevenness can be realized only if all steps in the ranked distribution have equal height. This distributional form is characterized by a linear descent of the representations and can be visualized as a *stepladder*. It will serve in the following as the *reference for complete unevenness* among a given number of types.

Stepladders are thus distributions *q* of the form $q_i=a_i$ with

3 $$\begin{aligned} a_i=\frac{s+1-i}{\frac{1}{2}s\cdot (s+1)}\quad \text {for}\ i=1,\ldots ,s \end{aligned}$$

They can also be conceived of as a finite arithmetic progression with increments (step-heights) of $a_i-a_{i+1}=1/[\frac{1}{2}s\cdot (s+1)]$. The relative step-heights are then given by $(a_i-a_{i+1})/a_1=1/s$ for $i=1,\ldots ,s$ as required (recall that $a_{s+1}=0$).

A distribution of maximal (or complete) unevenness can now be described for any given number of types by a stepladder characteristic, just as maximal (or complete) evenness is reached for a given number of types if all of them are equally represented. These two distributional characteristics set the limits between which the assessment of evenness or unevenness should operate. Since statements of unevenness as well as of evenness require consideration of at least two types, situations of monomorphism must remain indeterminate in both cases. In fact, a monomorphic distribution could be considered as a stepladder consisting of a single step and as a plateau consisting of a single type.

Diversity-based methods of assessing evenness cannot provide information on unevenness, since measures of diversity generally do not produce characteristic values that are associated with states of complete unevenness. Besides, the assessment of evenness basically relies on the proximity of a given type distribution to an ideal reference distribution, such as uniformity, and by this requires measures of distance between distributions in the first place. Measures of diversity, however, are aimed at properties of individual distributions rather than differences between such distributions, which makes them difficult to transform into meaningful distance measures. The following deliberations on measuring evenness/unevenness therefore take a different and largely diversity-independent route.

### Measures of Evenness/Unevenness

There are several ways to design measures that range between states of complete evenness and unevenness. Two approaches will be introduced in the following because they have intuitive appeal and demonstrate the possibility of looking at evenness from different perspectives. One approach is based on the distances of a type distribution from states of complete evenness and unevenness (the generic approach), and the other again uses the two distances but applies them to the distribution of step-heights in the ranked distribution as an indication of the deviation from states of complete evenness and unevenness (the step-height approach). We will start with an example from the generic approach, since this approach builds on perceptions that are more familiar from previous work on the topic. In a second step, a measure will be introduced that is based on the step-height approach.

#### The Generic Approach

Let *u*(*k*) denote a uniform distribution of *k* types (plateau of length *k*), and let *a*(*l*) be a stepladder of length *l*. Note that the stepladder consists of components

4 $$\begin{aligned} a_i(l)=\frac{l+1-i}{\frac{1}{2}l\cdot (l+1)}\quad \text {for}\ i=1,\ldots ,l\\\text{and}\ a_i(l)=0\ \text {for}\ i>l\end{aligned}$$

For the plateau, one has $u_i(k)=1/k$ for $i=1,\ldots ,k$ and $u_i(k)=0$ for $i>k$.

The distances of a type distribution *q* from the two reference distributions *u*(*k*) and *a*(*l*) can then be written as *d*[*q*, *u*(*k*)] and *d*[*q*, *a*(*l*)]. Among the many distance measures *d* between distributions, p-norm based distances (or simply p-distances) are probably among those most frequently applied. In the case of the distributions *q* and *a*, for example, they take the form $d[q,a(l)]=d_p[q,a(l)]=\big (\sum _i|q_i-a_i(l)|^p\big )^{1/p}$ with $p\ge 1$. In the above-referenced paper of Bulla, the distance $d=\frac{1}{2}d_1$ is used, and only distances *d*[*q*, *u*(*s*)] are considered for which *s* equals the number of types realized in the distribution *q*.

As the present concept is built on classifying and quantifying the closeness of a given distribution either to a plateau or to a stepladder, the primary task is to determine and compare the distances from the nearest plateau and the nearest stepladder. One thus needs to know the values $\pi :=\min _{k=1,2,\ldots }d[q,u(k)]$ and $\lambda :=\min _{l=1,2,\ldots }d[q,a(l)]$ as the minimal distances of the distribution *q* from a plateau *u* and a stepladder *a*, respectively. $\pi$ was previously used by Gregorius (1990) for the measurement of evenness. Equality of $\pi$ and $\lambda$ can then be considered to be a transition situation, a turning point, or a state of indeterminacy between evenness and unevenness.

The minimization of distances once more emphasizes the fact that ever larger distances from states of complete evenness should not *a priori* be considered as increasing closeness to states of complete unevenness, especially if these states are not explicitly defined. The same reasoning applies in the reverse direction, i.e., increasing distance from states of complete unevenness does not necessarily imply higher evenness, even though states of maximal evenness are properly defined. It is, in fact, conceivable that *q* may undergo changes along which their distances from both plateaus and stepladders increase, so that the two distances need not be negatively correlated. This, however, has no bearing on the assessment of evenness, since it is determined by the relation between values of two distances rather than by their absolute values. Therefore the quotient

$$\begin{aligned} e1:=\frac{\lambda }{\pi +\lambda } \end{aligned}$$

would be a consistent measure of evenness in the sense that *e*1 becomes 1 for complete evenness of *q* [when it equals one of the *u*(*k*)], and it becomes 0 for complete unevenness of *q* [when it equals one of the *a*(*l*)]. The two minimization processes also guarantee that *e*1 is independent of the number of types and prevent discontinuous changes in *e*1. Such changes occur when rare types are added to *q*, as may easily happen for increasing sample size.

For purposes of geometric illustration, it might be helpful to picture *e*1 as defining a surface above a frequency simplex, as is done in Fig. 4 in Sect. 3.2.

To find limits for the plateau and stepladder length below which the respective minimal distances $\pi$ and $\lambda$ must be realized, consider the minimal frequency $q_s$ in *q* and recall that $q_s\le 1/s$. For a plateau of length *k* with $1/k\le q_s$ (and thus $k\le s$), one obtains $2(1-x)$ as its $d_1$-distance from *q*, where $x=s/k$. The distance thus increases with increasing *k*, so that the minimal distance $\pi$ must be realized for a plateau of length *k* with $k\le 1/q_s\le s$. Along the same line of thought, consider that the maximal frequency of the stepladder is $1/\frac{1}{2}(l+1)\le q_s$. Since $q_s\le 1/s$, this implies $l\ge 2s-1$. The $d_1$-distance of this stepladder from *q* is $2(1-x)$, now with $x=\sum _{i=1}^s a_i(l)$, so that the distance increases with *l*. The minimal distance $\lambda$ must therefore be realized for a stepladder of length *l* with $l\le 2s-1$.

##### The Significance of Monomorphism

The index *e*1 is not defined for $\lambda =\pi =0$. Yet, for distributions of at least two types (polymorphism), $\pi =0$ cannot be realized simultaneously with $\lambda =0$, since this would imply that the distribution is a plateau as well as a stepladder. Coincidence of both, however, could only be conceivable for monomorphism, in which case the stepladder would consist of a single step. Hence, $\lambda +\pi >0$ always holds for polymorphic distributions. Monomorphism, in contrast, can be argued to be excluded from evenness deliberations, since any assessment of evenness or unevenness requires the comparison of different objects. Yet, especially for L-shaped distributions with one highly dominant and several rare types (minor polymorphism), the vicinity to monomorphic states is inevitable. Therefore, distances from monomorphism gain significance independently of its evenness/unevenness interpretation.

That consideration of monomorphism is actually essential, becomes evident when realizing that for a distribution approaching monomorphism (such as the geometric distribution in Eq. (1) with its parameter *x* approaching 0), the minimal distances $\pi$ and $\lambda$ are both obtained for the same limiting distribution, namely monomorphism. Hence, $\pi =\lambda$ and thus $e1=0.5$ ultimately hold, which is in complete conceptual accordance with the fact that no decision can be made in favor of evenness or unevenness at this limit. Furthermore, $e1=0.5$ continues to hold until the distribution includes types with sufficient representation to recognize tendencies towards either evenness or unevenness. This holds for all appropriate distance measures (including the $d_p$’s). It is this property that confirms the conceptual consistency of the method underlying the design of *e*1.

The situation of minor polymorphism will be returned to in more detail in Sect. 3.2 for suitable example distributions.

#### The Step-Height Approach

Another perspective of looking at evenness is less conventional but still intuitively obvious. It focuses on step-heights in the ranked distribution and their variability as an indicator of evenness. Step-heights are given by $q_i-q_{i+1}$ for the individual types, and since their sum equals $q_1$, one obtains for the distribution of step-heights the relative quantities $h_i:=(q_i-q_{i+1})/q_1$. With a stepladder *a*(*k*) of length *k*, step-heights are all the same, i.e., $h_i=h_i'(k)=1/k$ for $i=1,\ldots ,k$ and $h_i'(k):=0$ for $i>k$. For a uniform distribution (plateau), all step-heights are zero with the exception of the one that marks the end (or length) of the plateau. The p-distance of the step-height distribution *h* from the step-height distribution $h'(k)$ then reads

$$\begin{aligned} d_p[h,h'(k)]=\Big (\sum _i|h_i-h_i'(k)|^p\Big )^{1/p}=\Big (\sum _{i>k}h^p_i+ \sum _{i=1}^k|h_i-(1/k)|^p\Big )^{1/p} \end{aligned}$$

(recall that $h_i=0$ for $i>s$).

As mentioned above, the step-height distribution of plateaus, denoted by $h''(k)$, is degenerate in the sense that for a plateau *u*(*k*) of length *k*, the *k*-th component equals 1 while all other components are zero, i.e., $h''_i(k)=1$ for $i=k$ and $h''_i(k)=0$ for $i\ne k$. Hence, the $d_p$-distance, for example, of the step-height distribution *h* from the step-height distribution of a plateau of length *k* becomes

$$\begin{aligned} d_p[h,h''(k)]=\big ((1-h_k)^p+\sum _{i:i\ne k}h_i^p\big )^{1/p} \end{aligned}$$

For $p=1$, this reduces to $d_1[h,h''(k)]=2(1-h_k)$. Also recall that $d_1\ge d_p$.

Following the same construction principle as before, i.e., determining the position of type distributions between the closest stepladders and closest plateaus, type distributions are now to be replaced by their corresponding step-height distributions. The appropriate distances are then $\pi _h=\min _k d[h,h''(k)]$ and $\lambda _h=\min _k d[h,h'(k)]$, so that the evenness index becomes

$$\begin{aligned} e2=\frac{\lambda _h}{\pi _h+\lambda _h} \end{aligned}$$

with $e2=0$ only if *q* is a stepladder (complete unevenness), and $e2=1$ only if *q* is a plateau (complete evenness). It is again straightforward to show that $\lambda _h$ is realized for some $h'(k)$ with $k\le s$. The same holds for $\pi _h$ since $\pi _h=1-\max _k h_k$.

It is readily verified that the explanations on the significance of monomorphism given for the generic approach to *e*1 (Sect. 3.1.1) apply identically to the step-height approach to *e*2: for distributions strongly but not completely concentrated on a single type (minor polymorphism), one has $e2=0.5$. Further details are presented in Sect. 3.2, including illustrations of *e*2 as an evenness surface above the frequency simplex.

### Illustration of the Evenness Indices

General characteristics of the new evenness/unevenness measures can be demonstrated with the help of graphical representations of evenness. These include “evenness surfaces” that are drawn for the highest-dimensional case that is geometrically representable, namely for $s=3$ types, and “evenness curves” that follow one-dimensional transects (or lines) through the frequency simplex for any number of types. The following demonstrations are based on *p*-distances of order $p=1$ (i.e., $d=d_1$), because these are familiar from and allow comparison with the above-cited earlier approaches to the assessment of evenness and because they operate on untransformed differences between type representations.

All distributions that consist of at most *s* types form the frequency simplex $S_{s-1}$, an $(s-1)$-dimensional subset of *s*-dimensional real space (the one dimension is lost because each frequency $q_i$ equals 1 minus the sum of the others). The simplex $S_{s-1}$ has one plateau of length *s* in its center, and plateaus of shorter length are located on its edges. For *s* of any size, the simplex $S_{s-1}$ has $s!= s\cdot (s-1)...\cdot 2$ internal stepladders with *s* types, and stepladders with fewer types are located on its edges.

**Fig. 1:**
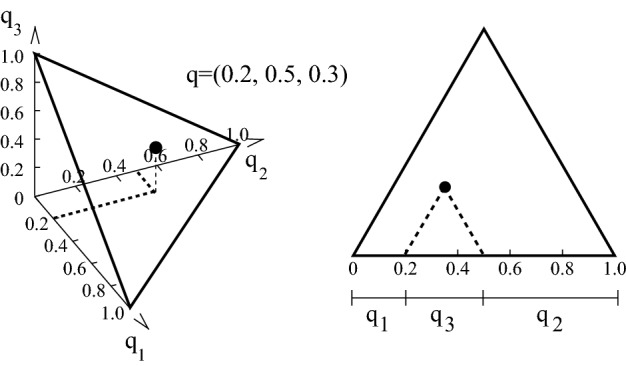
The frequency simplex $S_2$ as the set of all frequency distributions with at most $s=3$ types. Left panel: $S_2$ embedded in 3-dimensional real space ${\mathbb {R}}^3$. Right panel: Equivalent representation of $S_2$ as viewed from the origin (0,0,0) of ${\mathbb {R}}^3$. As an example, the distribution $q=(0.2,0.5,0.3)$ lies at the black dot

**Fig. 2:**
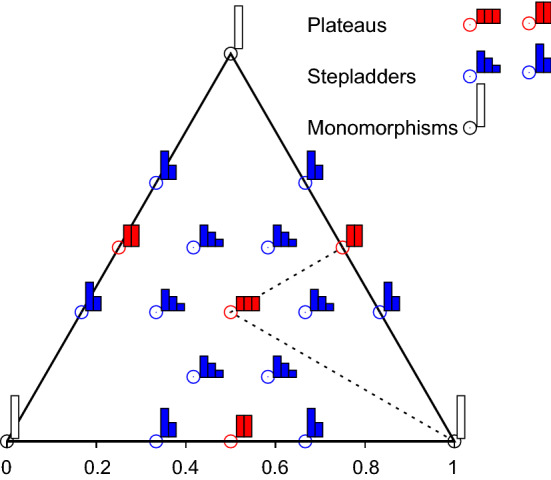
Special distributions of the simplex $S_2$. Of the four plateaus (red), the plateau $(1/3,\,1/3,\,1/3)$ of length 3 lies at the center of the triangle and each of the three plateaus $(1/2,\,1/2,\,0)$, $(1/2,\,0,\,1/2)$ and $(0,\,1/2,\,1/2)$ of length 2 lies at the center of one of the edges. Of the 12 stepladders (blue), the six stepladders of length 3 specified by $(3/6,\,2/6,\,1/6)$ and its five permutations surround the central plateau, and two of the six stepladders of length 2 specified by $(2/3,\,1/3,\,0)$ and its five permutations flank the plateau of length 2 on each edge. Each of the three vertices represents one of the monomorphisms (1, 0, 0), (0, 1, 0) and (0, 0, 1). The frequencies of all distributions within the wedge (dotted lines) are ranked in descending order, i.e., $q_1\ge q_2\ge q_3$. (Color figure online)

**Fig. 3:**
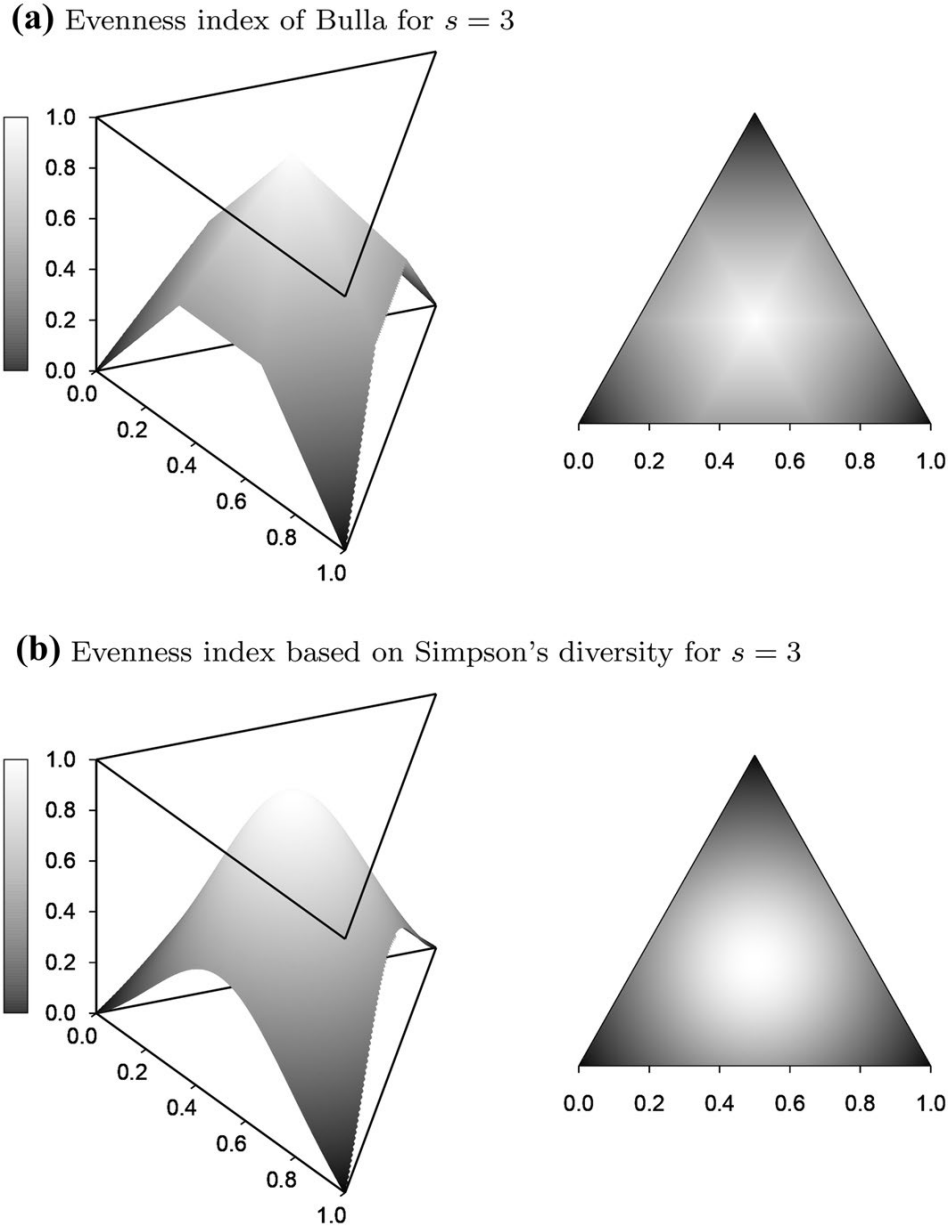
Surfaces of two widely used indices of evenness graphed over the frequency simplex $S_2$ for $s=3$ types: **a** Surface of the evenness index of Bulla (1994) seen from an upper viewpoint (left panel) and projected onto $S_2$ (right panel). **b** Surface of the evenness index based on Simpson’s diversity (Simpson 1949) seen from the same upper viewpoint (left panel) and projected onto $S_2$ (right panel). Color scale: from black for index value 0 to white for 1

**Fig. 4:**
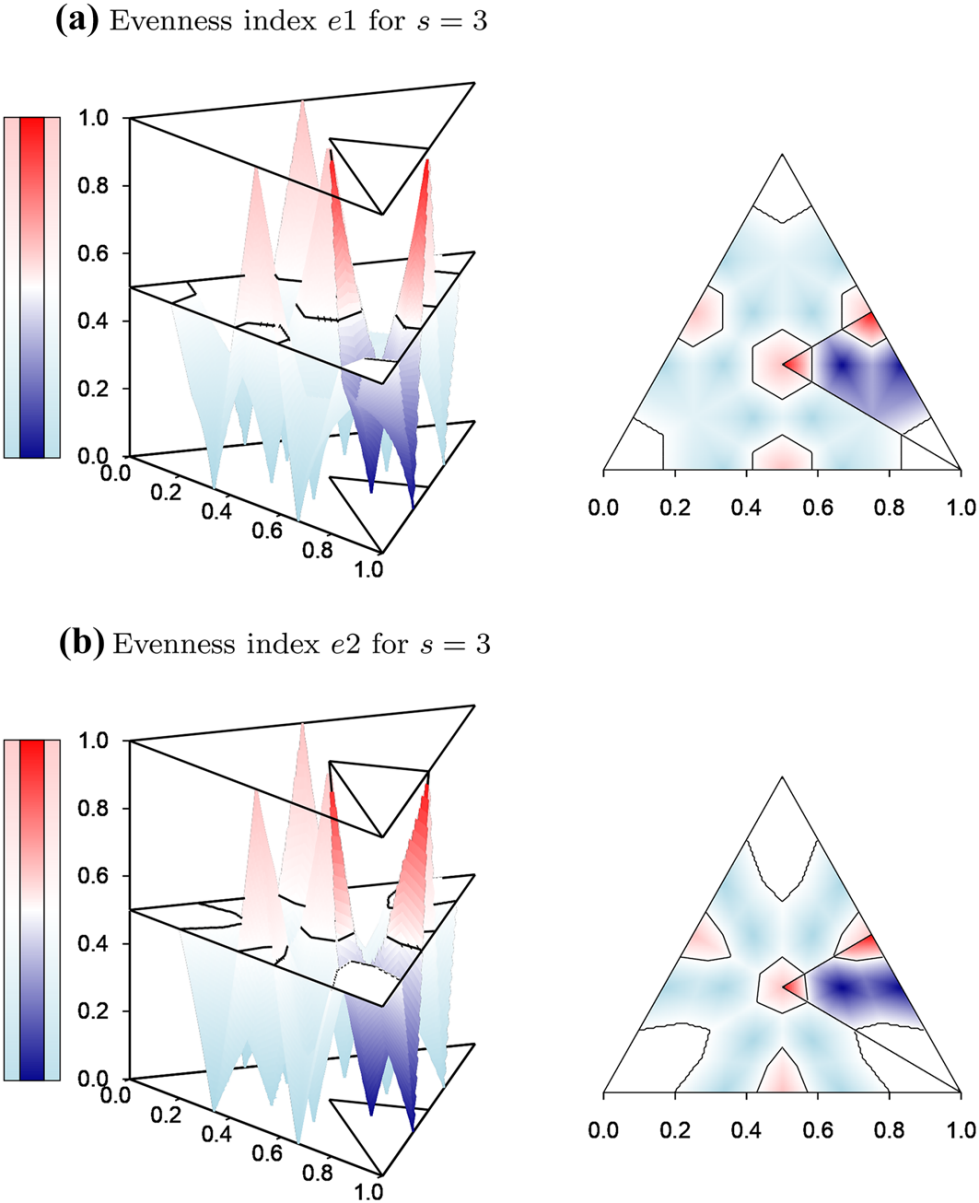
Evenness surfaces of the new measures *e*1 and *e*2 for $s=3$ types graphed above the simplex $S_2$. **a** *e*1-surface seen from an upper viewpoint (left panel) and projected onto $S_2$ (right panel). **b** *e*2-surface seen from the same upper viewpoint (left panel) and projected onto $S_2$ (right panel). Color scale: blue for evenness 0 to white for 0.5 to red for 1. In both projections (right panels), only the sector of the simplex is fully colored for which the frequencies of the distributions are ranked in descending order $q_1>q_2>q_3$; the rest of the simplex is shown in paler colors. The positions of the extreme values coincide for *e*1 and *e*2. In the left panels, the four peaks for which $e1=e2=1$ holds correspond to the four plateaus, and the low points for which $e1=e2=0$ holds correspond to the twelve stepladders (see Fig. 2). The indeterminacy of evenness/unevenness at the three monomorpic vertices is reflected by their evenness values of $e1=e2=0.5$; this indeterminacy is maintained as the two other types begin to appear at low frequencies, visible as the large white shelves at the corners. Between the extreme points, the contours of the *e*1- and *e*2-surfaces differ. Most prominent in the right panels are the narrower peaks, the wider low points and the larger shelves around monomorphism for *e*2 as compared to *e*1. (Color figure online)

Evenness can be visualized for $s=3$. The corresponding simplex $S_2$ can be drawn as a two-dimensional triangle on the plane (Fig. 1). The one plateau of length 3 is located in the center, surrounded by the six stepladders of length 3. The three plateaus and six stepladders of length 2 lie on the edges of the triangle, and the three monomorphisms are at the corners (Fig. 2). Evenness can be plotted vertically above each distribution in the triangle. When viewed from a suitable angle, the resulting three-dimensional evenness surface demonstrates the main characteristics of evenness (see Figs. 3 and 4).

To demonstrate the differences between the new measures and the evenness indices of Bulla (1994) and Simpson (1949), these two indices are graphed as surfaces over the simplex $S_2$ for $s=3$ in Fig. 3. Both are seen to assume their maximum of 1 only for the plateau $(1/3,\,1/3,\,1/3)$ of length 3 at the center of the simplex. Both ignore the distributions on the three edges of the simplex, where one of the types is of frequency 0, even though each edge contains a plateau of length 2 for the other two types and thus a point of highest evenness for $s=2$. This confirms their dependence on the inestimable number of types *s* (Pielou 1969). Moreover, Bulla’s index approaches its minimum of 0 as distributions approach monomorphism at the corners of the simplex, for example the *L*-shaped distributions mentioned above. Simpson’s index assumes its minimum of 0 there. Their assessment of monomorphism as minimal evenness contradicts the conclusion drawn above that monomorphism is actually of indeterminate evenness.

The measures *e*1 and *e*2 show similar behavior that is very different from the indices of Bulla and Simpson, as is demonstrated for $s=3$ by the evenness surfaces graphed over the simplex $S_2$ in Fig. 4. Red shading marks distributions with a tendency toward evenness, in that they are closer to a plateau of any length than to a stepladder of any length. Blue shading marks distributions with a tendency toward unevenness, in that they are closer to a stepladder than to a plateau of any length. Both measures have peaks (maximal turning points) of evenness 1 not only for the plateau $(1/3,\,1/3,\,1/3)$ at the center of the simplex but also for the plateau of length 2 that lies on each of the three edges of the simplex. Both measures also have minimal turning points of evenness 0 within the interior of the simplex, namely for the six stepladders with three types, as well as on each edge for the two stepladders with two types. The large ”shelf” of evenness 0.5 (white) surrounding each of the three monomorphic corners of the simplex indicates that evenness is indeterminate not only for complete monomorphsim but also as individuals of the other two types begin to appear, as proven above for arbitrary number of types.

The differences between *e*1 and *e*2 are easy to see in the right panels of Fig. 4, in which only those distributions with frequencies ranked in descending order are graphed in full color. Comparing the proportion of distributions with evenness greater than 0.5 (red shading around the peaks), *e*1 assigns more distributions a tendency toward evenness than *e*2. In like manner, comparing the proportion of distributions with evenness less than 0.5 (blue shading around the low points), *e*1 assigns more distributions a tendency toward unevenness than *e*2. This is balanced by the higher proportion of distributions of indeterminate evenness 0.5 (white shelves) for *e*2 than for *e*1. Thus for $s=3$, *e*1 is more definitive about evenness than *e*2. A reason for this is proposed at the end of this section.

It is more difficult to see whether this difference between *e*1 and *e*2 is maintained when distributions have more than three types, since evenness can no longer be drawn in three dimensions as a surface over a two-dimensional simplex. It is, however, possible to compare *e*1 and *e*2 as ”evenness curves” along one-dimensional transects (lines) through higher-dimensional simplices. For example, for two distributions *q* and $q^\prime$, the set of linear combinations $(1-x)\cdot q+x\cdot q^\prime$ forms a line of distributions through the simplex from *q* to $q^\prime$, where the parameter *x* runs from 0 to 1. An evenness curve can then be plotted as a function of *x*.

Problems in picturing evenness arise in particular as the number of types increases while the dominant type remains at properly positive representation. This can be demonstrated most efficiently with the help of L-shaped distributions when considering them as a linear combination of a completely uniform distribution (with proportion *x*) and a distribution consisting of a single type (with proportion $1-x$). In formal terms, L-distributions then become straight lines $q=x\cdot u(s)+(1-x)\cdot u(1)$ connecting the vector *u*(1) with *u*(*s*) in the frequency simplex. The linearity of p-distances *d* then implies $d[q,u(s)]=(1-x)\cdot d[u(s),u(1)]$. When applied to Bulla’s index (with $d=d_1$), this yields *x* for the index and is thus independent of the number of types (compare Fig. 3). Hence, the index tends to 0 with *x* tending to 0, which again confirms the inconsistency of the index. In contrast, Simpson’s evenness index $N_2/s$ (as well as $(N_2-1)/(s-1)$) tends to 0 with increasing *s* and for constant *x*, which reveals another kind of inconsistency of this diversity-based type of evenness measure.

**Fig. 5:**
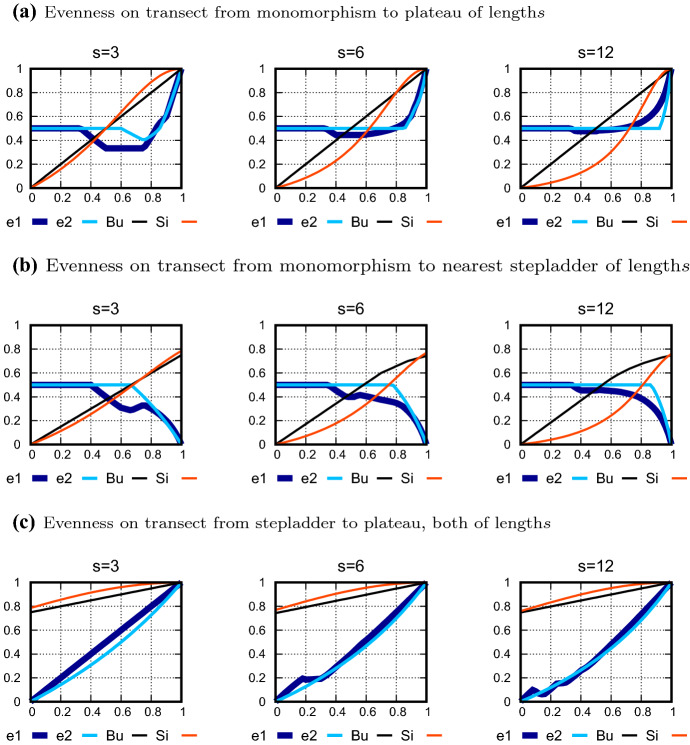
Evenness along transects through the frequency simplex $S_{s-1}$: Evenness is graphed along transects specified by the linear combinations $(1-x)\cdot q+x\cdot q^\prime$ of two distributions *q* and $q^\prime$, where the parameter *x* ranges from 0 to 1 and the *p*-distance $d_1$ is used. The new measures *e*1 and *e*2 and, for comparison, the indices of Bulla (Bu) and Simpson (Si) are shown. **a** L-shaped distributions from monomorphism $(x=0)$ to the plateau of length *s* $(x=1)$, where $s=3$ (left), 6 (center), and 12 (right); **b** from monomorphism $(x=0)$ to one of the nearest stepladders with *s* types $(x=1)$ for $s=3,6,12$ [e.g. from (1, 0, 0) to $\left( {3/6,\,2/6,\,1/6} \right)$]; **c** from a stepladder with *s* types $(x=0)$ to the plateau of length *s* $(x=1)$ for $s=3,6,12$. For $s=3$, the evenness along these transects corresponds to the coloring in the right panels of Fig. 4

By viewing evenness curves along analogous transects through simplices with different numbers of types, such as from the plateau of length *s* to a stepladder of length *s*, it can be seen how evenness develops as the number of types increases. Figure 5 shows evenness curves over three sets of transects, each set with analogous starting and ending points for $s=3,6$ and 12 types:

Figure 5a shows the transect through the simplex $S_{s-1}$ from a monomorphic corner ($x=0$) to the plateau of length *s* at the center of the simplex ($x=1$). All distributions on this transect are L-shaped distributions, as specified in Eq. (2). The most striking impression is that *e*1 leaves the ”shelf” of indeterminacy (0.5, as in Fig. 4) that starts at monomorphism at approximately the same value of *x* for all three *s*. In contrast, *e*2 leaves its shelf later than *e*1 and extends it farther, the larger *s* becomes. Thus the ascent of *e*2 to its peak of 1 at the central plateau ($x=1$) is steeper than for *e*1 for all *s*, and it becomes even steeper as *s* increases. This confirms the conclusion from the projections in the right panels of Fig. 4 that for $s=3$, the area around the central plateau with tendency toward evenness is larger for *e*1 than for *e*2. For intermediate values of *x*, both measures dip down into unevenness, but this tendency becomes less pronounced for *e*1 and it disappears for *e*2 as *s* increases. These characteristics contrast with the indices of Bulla and Simpson, which show the full range of evenness from 0 to 1 along the transect, While *e*1 and *e*2 range only between mild unevenness and evenness of 1.

Figure 5b shows the transect from a monomorphic corner ($x=0$) to one of the stepladders with *s* types that is closest to this corner ($x=1$). *e*1 and *e*2 stay on their shelves for about the same stretch of *x* as they did in Fig. 5a, with *e*2 remaining indeterminate much longer than *e*1. After leaving their shelves, both measures tend toward unevenness. As *s* increases, the descent of *e*2 to its low point of 0 at the stepladder ($x=1$) begins later and is therefore steeper than for *e*1. While *e*1 and *e*2 range from indeterminate to complete unevenness along the entire transect, the indices of Bulla and Simpson start at 0 and increase to high evenness.

Figure 5c shows the transect from a stepladder with *s* types ($e1=e2=0$ for $x=0$) to the plateau of length *s* ($e1=e2=1$ for $x=1$). The curves become more similar as *s* increases, but they differ in that *e*2 is smooth while *e*1 show bumps for small *x*. These bumps occur when the transect comes closest to stepladders or plateaus of different lengths. While *e*1 and *e*2 cover the full range from 0 to 1, the indices of Bulla and Simpson show high evenness along the entire transect.

The evenness curves in Fig. 5 confirm the impression given by the evenness surfaces in Fig. 4 that *e*1 is more sensitive in its assessment of evenness than *e*2. *e*2 retains the state of indeterminacy (0.5) over a much longer distance from monomorphism than *e*1, while *e*1 decides at a much shorter distance to show a tendency toward evenness or unevenness. An apparent reason can be seen in the fact that in the step-height approach, stepladders appear as uniform distributions and plateaus appear as degenerate distributions with a single non-zero component. Both distributional characteristics allow for fewer adjustments in the minimization of distances than is the case for the original frequencies on which *e*1 is based. Thus *e*1 allows a finer adjustment of its tendencies than *e*2 or, in other words, *e*1 is more decisive in its assessment of evenness.

### Variable Differences Between Objects

Especially when aspects of evenness are to be considered for the joint distribution of multiple traits, one quickly arrives at the situation where all trait combinations are unique and evenness considerations are without substance. Indeed, in this case each type occurs exactly once, so that evenness would always be maximal under the perspective of discrete types. Yet, the various trait combinations may differ more or less for the states of the individual traits, so that evenness aspects are to be supplemented by variable differences among the entities of interest (for a typical data set, see Table 1). These compound traits are determined for single objects, and they include phenotypic, functional, taxonomic, genetic, environmental, or geographic characteristics, all of which may differ to variable degrees among the objects. Genealogical or phylogenetic traits are different in that they specify traits for pairs of objects (e.g. degree of relationship) rather than individual objects.

Indeed, consideration of variable differences in analyses of variation, and diversity in particular, has received much attention during the last years. Essentially, this is due to the availability of techniques for the identification and assessment of complex traits, particularly in molecular genetics. Applications especially in ecology frequently appear under the notion of functional diversity. This will be taken up in the next subsection with emphasis on functional evenness.

Even though the concept of evenness has been developed so far only for discrete traits, it can be consistently extended to arbitrarily complex traits for which differences between the trait states are defined. To this end, it must be realized that the contribution of the types (complex trait states) of individual objects to a collection’s evenness cannot be assessed independently of their difference from the types of the other objects in the collection. Therefore, at the outset, contributions to a collection’s evenness are determined by the trait differences between the two objects in every *pair of objects* as well as by the abundances of such pairs in the collection. Thus the entities of analysis are now pairs.

The challenge is then to see whether and how pairs of objects or types, their abundances and their differences can be packaged into a conceptually consistent framework of evenness measurement. Apparently, a more comprehensive definition of the term “representation” is needed. Continuing and generalizing the above demonstrations suggests that *representation be generally conceived as a prescription for assignment of real values to entities, where the values measure each entity’s share in the totality and by this specify a distribution for the entities*. The representation of a subset of entities consequently equals the sum of the representations of the included entities. Evenness then describes the situation in which the representations of the entities conform with a uniform distribution, and this applies in particular when the entities are pairs of objects (or types).

There are several ways in which pairs can be characterized as entities that yield a distribution. Ignoring differences for the time being, pairs may be defined, for example, by mating events and characterized by mating types. The frequencies of realization of these mating types then specify their representations. Instead of mating events within species, encounters between two individuals of the same or of different species affiliation could be recorded, so that pairs of species constitute the entities under consideration and the frequencies of encounter specify their representations. The latter includes the possibility of forming all potential species pairs with representations given by the product of the involved species abundances. In these cases, the respective frequency or abundance products reflect the share that each entity has among the totality of entities. Evenness assessment is thus legitimate, and the above indices of evenness/unevenness apply identically. Yet, the explicit introduction of type differences require more consideration of pair characteristics, as will be detailed in the following.

#### Evenness for Variable Differences (Variational Evenness)

Inclusion of differences in performance among entities into evenness deliberations are frequently treated in terms of “functional evenness”. As will be shown later on, current methods of quantifying functional evenness and its relatives apparently pass over the distinction that can be made between the following three levels of variation: (1) the distribution of differences, (2) the variability of differences, and (3) the significance of differences as representations of pairs in a collection of pairs. As to level (3), it should be recalled (see above) that identifying and quantifying differences involves two objects in each case and therefore basically rests on the evaluation of pairs and their properties. These properties do not *per se* reflect a share that the pairs have in the totality of pairs. In fact, pair differences cannot be a subject of studies of evenness unless they can be explicitly shown to be part of a representation of single pairs among all pairs and thus establish a pair distribution.

$\bullet$ Level (1) clearly addresses evenness, as complete evenness of the distribution is indicated by equal representation of all differences. In essence, differences adopt the role of types here, making the numerical values of the differences irrelevant. The numerical values can still be used in the construction of cumulative distribution functions as helpful characterizations of difference distributions. High evenness can be expected, for example, in the absence of any advantages of functional interactions among community members for the trait under consideration. The trait could thus be assessed as functionally neutral. In contrast, decreasing evenness could be caused, for example, by functional superiority of interactions preferably among individuals of similar trait expression.

$\bullet$ Level (2) is relevant when pairs are the entities of interest and where these entities are characterized by pair differences. Hence, with this focus, problems of dispersion and especially of variance of differences are to be studied. Distributional aspects are not directly concerned, so that evenness is not an immediate issue. Nonetheless, the absence of variability as insinuated by the term evenness could lead to the idea that there is complete evenness if there is no variation in differences. Apparently, if differences of zero were included, this would imply the meaningless conclusion that variation in differences is absent (and evenness is maximal) if all differences are zero. Differences of zero are to be excluded from the analysis anyway.

Under this restriction, variances of differences are used especially in phylogenetic analyses to quantify notions of regularity of phylogenetic structure [for a compilation see Tucker et al. (2017, Appendix S1)]. In this context, the term “regularity” is preferred over “evenness”. Complete regularity is then realized for zero variance and thus for equality of all non-zero differences. Abundances are thus not regarded here, so that complete regularity is stated despite arbitrarily variable abundances. It should also be realized that complete regularity in fact implies a change in category of trait, in that only discrete traits can display complete regularity.

In many cases, the entities of dispersion analyses for variable differences are specified by individuals or types rather than pairs of these. For example, Euclidean representation of data points together with Euclidean distances (Gower 1971) is often applied, especially for complex multi-trait characters [e.g. Villéger et al. (2008), Pavoine et al. (2009)]. The pertaining analyses of variation again belong to the class of dispersion studies which, however, are subject to the restriction that equality of differences between all data points cannot be realized if the number of such points exceeds the number of traits (dimensions in the Euclidean space). The conceptual implications of this restriction for evenness considerations can be circumvented only by reducing analyses to selections of special pairs, as will be returned to later in connection with minimal spanning trees (MSTs). In other words, *without explicit reference to pairs as conceptual entities, attempts to describe and quantify evenness in dispersion may be problematic*.

A general drawback of using variances in evenness or regularity analyses is again to be seen in the problem of specifying maximal variance (if it exists at all) in terms of minimal evenness (maximal unevenness) or in terms of minimal regularity (maximal irregularity). As indicated earlier, without such specifications, non-zero variances have no definite qualitative interpretations.

$\bullet$ Level (3) can be approached by recalling that any given representation of entities can be transferred into a new representation of the same entities by applying a non-negative transformation to the initial individual representations. After normalization, if desired, the resulting representation appears as a modification of the initial distribution generated by the transformation.

This applies to pair entities as well and therefore relates to level (2). For example, if the initial pair representations are denoted by $q_{i,j}$ for each pair (*i*, *j*) of objects *i* and *j* with associated difference $d_{i,j}$, then a modification of the $q_{i,j}$’s that considers the differences $d_{i,j}$ is provided by the transformation $q_{i,j}\cdot d_{i,j}$, or as relative representation, $q_{i,j}^*=q_{i,j}\cdot d_{i,j}/\sum _{k,l} q_{k,l}\cdot d_{k,l}$ (see Table 1). In most applications, the initial pair representations are of the form $q_{i,j}=q_i\cdot q_j$, with $q_i$ and $q_j$ as the object representations realized in the initial marginal distribution (see e.g. Rao (1982) and the numerous applications of his average difference $\sum _{k,l} q_k\cdot q_l\cdot d_{k,l}$ not just in the field of functional diversity).

**Table 1:**
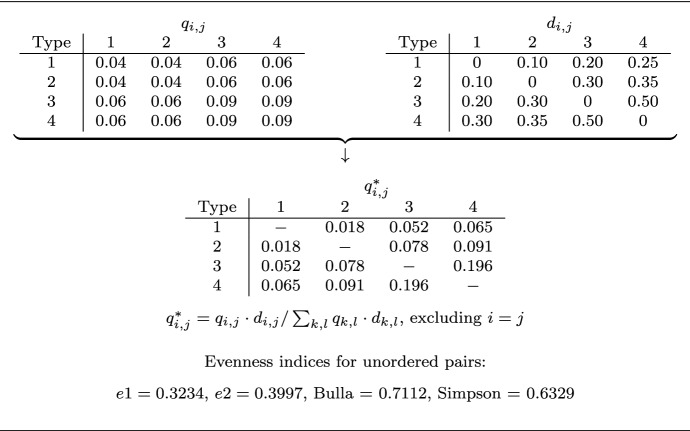
Evenness with variable type differences

$q_{i,j}=$ pair abundances, $d_{i,j}=$ pair differences, $q_{i,j}^* =$ modified representations considering pair differences, $e1 =$ generic and $e2 =$ step-height approach to evenness, Bulla = Bulla’s evenness index, Simpson = evenness index based on Simpson’s diversity

Differences *d* are chosen to increase with abundances *q*, so that the unevenness in the abundances is expected to be enhanced by the differences. Despite many equal abundances, this leads to values of *e*1 and *e*2 below 0.5, thus indicating tendencies towards unevenness. In contrast, the indices of Bulla and Simpson suggest a distribution of comparatively large evenness

With this transformation, however, one arrives at pair representations for which the identity pairs (*i*, *i*) prevent the attainment of a state of complete evenness, since $d_{i,i}=0$ implies that $q_{i,i}^*=0$, with the result that equality of all $q_{i,j}^*$ cannot be realized. As pointed out earlier, it is therefore essential to generally *exclude identity pairs from analyses of variational evenness*.

In the latter context, it is possible to assume complete evenness for the initial representation (all $q_{ij}$ identical for $i\ne j$) so as to uncover the effect of differences on representation. For the above example, the pair representations modified by difference would then take the form $d_{i,j}/\sum _{k,l} d_{k,l}$ for ${i\ne j}$. Even though this gives the impression that the differences themselves appear as pair representations, it should be recalled that it owes this claim only to a particular underlying initial pair representation [for a compilation of relevant indices, see Scheiner (2019)]. Conversely, the effect of the initial representation on the evenness of the modified representation can be considered by setting all differences between different types equal.

Occasionally, for the pair representations $q_i\cdot q_j\cdot d_{i,j}$, the impression is conveyed that complete evenness is reached only if all types have equal representation and all differences between different types are equal. This claim is not true, as can be demonstrated for three types by choosing arbitrary values for $d_{1,2}$ as well as for all three $q_i$’s ($q_1+q_2+q_3=1$) and setting the remaining differences to $d_{1,3}=d_{1,2}\cdot q_2/q_3$ and $d_{2,3}=d_{1,2}\cdot q_1/q_3$. Moreover, the initial claim is intrinsically meaningless, since equality of all differences is of relevance for evenness only in connection with pair distributions. Equality of differences therefore indicates complete evenness only if *a priori* all involved pairs are equally represented.

In summary, the above demonstrations reveal that when evenness analyses are to consider variable differences between types, only level (3) of variation with its modified pair representations is of relevance.

#### Special Cases

In some applications, only certain kinds of pairs are considered in evenness analysis. For example, trying to align species in a linear fashion, Villéger et al. (2008) extracted minimum spanning trees (MSTs) from their difference matrices and supplemented each MST-edge weight (difference) by the sum of the abundances of the two species defining the edge vertices. Pairs are thus specified by two species that flank an MST-edge whose pair representations are defined by the edge weight combined with the corresponding species abundances. Though this might appear as a poorly argued and partially indeterminate approach to the assessment of “functional diversity” components (Kosman et al. 2021), its principle fits into the present pair representation framework and can therefore be used in evenness analyses. Yet, as Kosman et al. (2021) pointed out, because of the conceptual vagueness (multiple MSTs) and ambiguity of the general approach of Villéger et al., application of their method of measuring functional evenness is not to be recommended.

Pair differences also serve as the basis for characterizing individuals or types by neighbor relations, such as the smallest difference or the average difference of one individual from individuals of another type (in the present terminology $\min _{j:j\ne i}d_{i,j}$ and $\sum _{j:j\ne i}d_{i,j}\cdot q_j/(1-q_i)$, respectively). The entities to be represented are then types and no longer pairs of types, and the character assigned to each type now depends on the other members of the community. The initial representation of the types is now provided by their abundances, for example, and the modified type representations are $q_i\cdot \min _{j:j\ne i}d_{i,j}$ and $q_i\cdot \sum _{j:j\ne i}d_{i,j}\cdot q_j/(1-q_i)$, respectively [for usage of the latter in measuring functional evenness, see e.g. Ricotta et al. (2014) or Scheiner (2019)]. Dispersion and evenness considerations are again distinguished by whether the focus is on variability in neighbor relations of types or on modification of type representations by neighbor relations, respectively.

Similar kinds of type representations can be applied to the assessment of structural diversity, where abundance and distinctness of types are the essential determinants of community structure (Gregorius and Kosman 2018). In connection with an appropriate index, this allows specification of structural evenness. High evenness in structure, however, is commonly considered as a situation that opposes natural systems, since these are generally characterized by higher degrees of complexity which, in turn, imply irregularity. As a matter of fact, irregularity and thus unevenness plays a major role in studies of community stability [see e.g. May (1974)]. This underlines the significance of the present indices *e*1 and *e*2 with their conceptual emphasis on the specification of unevenness.

Neighborhood relations may, however, also be viewed as sets of pairs, where the two individuals forming a pair are required to meet certain criteria, such as at least one being the nearest spatial neighbor of the other. The spatial distances of such neighbors then define their difference, and all pairs are given the same representation. Computation of evenness values would then provide information on the regularity or irregularity of neighbor relations. Stepladders as situations of complete unevenness then appear as states of complete irregularity of neighbor distances. Indeed, this is intuitive in the sense that all possible neighbor distances are realized and vary in equal measure, so that no clumping or partitioning occurs.

Scheiner et al. (2017) took an approach which could be considered as hybrid in that it combines two levels of entities, individual type and pair of types. In a first step, pair diversity is defined by Hill numbers applied to pair representations modified by differences. Next, the number of types effectively contributing to pair diversity is determined. The ideal situation underlying this effective number is characterized by pair frequencies obtained from the products of all (marginal) type frequencies, by equal type frequencies, and by equal (non-zero) differences. Their functional evenness index then results in the common fashion from division of the effective number of types by the total number. The effective number is a strictly increasing function of the pair diversity, and it is less than or equal to the total number of types with equality only if the pair representations are given by the above ideal situation. Hence, to realize states of maximal evenness in the sense of the index of Scheiner et al., equality of pair representations is not sufficient, since this includes situations in which not all type combinations are represented (this observation will be returned to in the next subsection). The minimal value of the index is realized if only two types exist and these form the single pair. The present criticism of conceptual inconsistency with respect to states of complete unevenness applies accordingly.

#### The Role of Types in Measuring Variational Evenness

For *s* types realized in a set of pairs with positive representation (recall that identity pairs are excluded), the smallest number of such pairs is *s*/2 for even *s* and ${(s+1)/2}$ for odd *s*, and the largest number is ${s(s-1)}$. For any number of pairs within this range, equality of all pair representations and zero representation of the other pairs is possible. This would imply maximal evenness according to the present concept if pairs were considered as simple entities, ignoring the types of which they are composed. The above MST example belongs to this category, since there the edges and their weights but not the involved vertices are relevant.

Obviously, this is undesirable if the two types that make up each pair are explicitly defined. Then the observed number of types (with positive representations) is understood to determine ideas of variational evenness/unevenness in the first place, and pair representations follow this pattern in some sense. In particular, a pair distribution involving *s* types is considered to be a plateau only if all of the ${s(s-1)}$ possible type combinations (and not fewer) are equally represented. In this case, the associated type representations also are all equal ($=1/s$) and therefore form a plateau at the type level.

Stepladders, as states of complete variational unevenness, follow the same principle as plateaus in that all possible type combinations are required to be realized (with positive representations) and their representations show stepladder structure. Hence, at the pair level, stepladder distributions as well as plateaus are completely determined by the number of types involved. They specify the states of complete variational unevenness and complete variational evenness. Consequently, the length of the plateaus and stepladders involved in the minimization of distances from the observed distribution of pair representations are determined by the involved number *k*, say, of types.

For the lengths of the plateaus and stepladders to be considered in the minimization of distances, ordered pairs [with numbers ${k(k-1)}$] have to be distinguished from unordered pairs [with numbers ${k(k-1)/2}$]. Thus in the former case, lengths proceed in steps of ${k(k-1)}$ and in the latter case in steps of ${k(k-1)/2}$ with $k=2,3,4,\ldots$. Note that unordered pairs cannot be treated as symmetrically ordered, since the latter would exclude the existence of stepladders (in Table 1, evenness indices are determined for the unordered pairs resulting from the symmetrically ordered pairs). The resulting minima $\pi$ (or $\pi _h$) as well as $\lambda$ (or $\lambda _h$) enter the definition of the evenness indices *e*1 (or *e*2), as before. The indices could then be referred to as indicating variational evenness according to the generic and to the step-height approach, respectively.

## Concluding Remarks

The presently suggested indices of evenness/unevenness cannot be used to construct indices of diversity in the usual way by multiplying the former index by the number of types (richness) and subsequent transformation. This multiplicative decomposition of diversity indices relies on their interpretation as “effective numbers” of types and is conceived to almost be a principle of diversity measurement [see e.g. Eq. (1) in Tuomisto (2012)]. Yet, according to the present demonstrations, it is just this decomposition that implies conceptual inconsistency by not distinguishing clearly between the notions of evenness of type representations and concentration of mass to or dominance by a single type, for example.

The intrinsic reason could be seen in the fact that the evenness criterion for diversity cannot be simply reversed to address aspects of unevenness. In fact, the reversal would read “diversity decreases as the difference in frequency between two types increases while the sum of their frequencies remains the same”. As can be easily imagined, when applying this criterion repeatedly to a sequence of decreasing type representations, one would again arrive at a distribution that ultimately consists of a single type, which reinforces the above-argued inconsistency. The same line of reasoning applies to Bulla’s approach, in which the evenness criterion is essentially replaced by the distance from a uniform distribution. This justifies the conclusion that indices of this kind should be addressed as indicators of the absence of concentration of the overall mass to or dominance by a single type or, as argued above, more appropriately as indicators of relative polymorphism.

The evenness criterion of diversity therefore is not suitable for describing evenness in terms of equality and inequality in representation among types. Consequently, the present indices abandon any diversity orientation and focus solely on the complementary notions of equality (sameness) and inequality (differentness) of type representations. The fact that situations of high evenness are much less frequently observed than variable representations [see e.g. Mulder et al. (2004)] obviously calls for approaches which sharpen the focus on unevenness. In particular, this includes specification of the state of complete unevenness with the same precision and intuitive appeal as is familiar from evenness. Since the evenness criterion is conceptually tied to notions of diversity in terms of “effective numbers” of types, it is not surprising that it leads to ambiguous interpretations when identically applied to generalized aspects of evenness. The diversity-bound evenness criterion together with its implied idea of diversity decomposition should therefore be abandoned as a defining criterion of the general notion of evenness. The present deliberations and demonstrations provide alternatives.

Application of the concept of measuring evenness to variable differences raises the question of its relation to the measurement of dispersion. The latter addresses first and foremost the spread of measurements and by this again summarizes differences and abundances or other representations [for a conceptual treatment see Gregorius and Kosman (2017)]. It, however, provides no information on the variability of type differences and type representations in communities. This is well known for variances, for example, where the same variance can be realized in communities with many individuals varying strongly in difference and in communities with individuals largely differing by the same amount.

In other words, dispersion indices report the amount of variation but not its distribution. The latter is covered by indices of evenness for variable differences. Such indices, especially of the form discussed above, thus provide essential complementary and independent information on dispersion, which suggests that they be addressed as indicators of *dispersion evenness*. This feature of dispersion seems to be largely ignored, however, with the possible exception of a few indices listed in the paper of Scheiner et al. (2017).

Another evenness aspect that was already mentioned above with reference to phylogenetic characteristics is termed “regularity”. Tucker et al. (2017, p. 710) stated that “regularity metrics reflect evenness in the distribution of dissimilarity among species, ...” which is strongly reminiscent of dispersion evenness. Yet for example, the regularity indices referring to tree topology listed by Tucker et al. (2017, Appendix S1) are difficult to associate with differences among species but rather seem to relate to ideas of network symmetry and the like.

## Data Availability

No real data or other material was used.

## Code Availability

The software developed for calculation of the numerical example is available from Elizabeth M. Gillet.
